# Evaluating Adherence to Rapid Tranquilization Protocols in Psychiatric Emergencies: An Audit of a Tertiary Care Facility in Pakistan

**DOI:** 10.1192/bjo.2024.571

**Published:** 2024-08-01

**Authors:** Naima Gul, Asad Nizami

**Affiliations:** Institute of Psychiatry, Rawalpindi Medical University, Rawalpindi, Pakistan

## Abstract

**Aims:**

This audit assesses the adherence to and effectiveness of rapid tranquilization protocols in a tertiary care psychiatric facility in Pakistan, particularly focusing on the use of intramuscular (IM) haloperidol and promethazine. The evaluation also includes an analysis of how these practices align with the prescribed guidelines for managing psychiatric emergencies.

**Methods:**

A comprehensive retrospective analysis of patient records from January to December 2023 was conducted. The focus was on assessing the sequence of interventions (de-escalation techniques, oral medication, IM administration), medication choices, adherence to protocol steps, and documentation of patient monitoring post-administration. Descriptive and inferential statistical methods were applied to analyze the data.

**Results:**

Among 482 patient records:

The primary diagnoses included schizophrenia (44%), bipolar disorder (29%), and severe depression with psychotic features (27%). IM haloperidol and promethazine were predominantly used, with 68% of cases bypassing oral medication or de-escalation attempts. Only 60% of cases showed adherence to the recommended protocol steps, including assessment for medical causes and optimization of regular prescriptions. In 12% of cases, a second injection was necessary, with the interval between injections undocumented in 15% of these cases. Vital monitoring post-administration was not recorded in 30% of cases. Medication unavailability was an issue in 8% of aggressive cases. Protocol deviations included the omission of recommended pre-treatments, such as ECG for haloperidol and the lack of alternative options like buccal midazolam or inhaled loxapine.

**Conclusion:**

The audit reveals significant deviations from established guidelines in the rapid tranquilization process. The frequent omission of non-invasive interventions and the lack of consistent monitoring and documentation practices highlight areas needing immediate improvement. Training in de-escalation techniques, adherence to step-wise intervention protocols, and ensuring the availability of a range of medications are crucial. This study underscores the importance of aligning psychiatric emergency practices with established guidelines to ensure patient safety and effective treatment outcomes.

